# Modern Sociology

**Published:** 1898-12-10

**Authors:** 


### Modern Sociology.

### REFORM IN THE CRIMINAL LAW.

By Francis Scougall.

On a date so recent as October 17th, 1898, a late enactment of the Legislature came into active operation as a new law of criminal procedure, which will make a very great and, as I conceive, beneficial change in the conduct of cases brought before our Courts of Justice. The enactment in question is entitled "The Criminal Evidence Act," and it accords liberty to prisoners brought up for trial to enter the witness-box and give evidence in their own defence—as witnesses in no way implicated in the offences charged against them have alone been allowed to do hitherto. The Act also empowers a man's wife to state what she knows in his favour, and these concessions undoubtedly remove one of the greatest grievances that, among the lower orders especially, has been held to exist in the mode of criminal procedure, which has been the rule in our Law Courts up to the present month. The honour of passing this wise and just Act falls to Lord Halsbury, but the Lord Chancellors holding office for many years past have each and all sought to accomplish it. So long ago as in the year 1853 Lord Brougham was enabled to pass the Evidence Act, which allowed parties in Civil suits only to give testimony at the trial after the speech for the prosecution.

The present Act involves, of course, much more serious considerations in many ways, and it is therefore not surprising that one learned judge at least, and various subordinate law officers, object to it, and regret its having become operative as an unquestioned rule. There are, however, many names of undoubted weight among men who cordially approve of it, such as Sir Edward Clarke, Sir George Lewis, and others. The opponents of the Act bring forward various reasons for their objections to it, none of which appear to me well founded; for instance, they hold that it will cause an increase in the crime of perjury. According to much experience, the practice of perjury under the former régime was so extremely prevalent that it is not at all likely to be more general in the new arrangement. To a very large majority of the lower orders, truth is an unknown quantity—they consider lying to be one of the necessities of human existence, and when they are called into a witness-box it never occurs to them to have any other intention than to invent the best story they can, for the person in whose favour they wish to speak; or *vice versa*, if an enemy is in the dock, they will invent the most glaring accusations they can against him. The fact they they are upon oath is not a matter which, as a rule, impresses itself on their minds at all—certain words, which they do not trouble themselves to understand, have been dictated to them, and then they have been told to kiss the book—a ceremony which has practically no meaning for them of any kind. It is no doubt extremely likely that an unprincipled prisoner, defending himself in a witness-box, may commit perjury; but in cases where a counsel for the prosecution has thoroughly investigated the facts of his misdeed, and where an astute judge, accustomed to unravel the intricacies of human error, is critically watching every word of the prisoner's utterances, his possible perjury is not at all likely to impede the course of justice. The same may be said of another objection to the Act raised by its opponents. They say that any accused persons who are really innocent might, when giving evidence, be so nervous and confused in their statements as to give a false colouring to their facts, and so cause themselves to be found guilty when they did not deserve it. I have a higher opinion of the acumen of our judges and barristers than to consider this likely or even possible. Another result of the Act is said to be in the strong probability that husbands and wives will be found to accuse one another, as before the passing of this Act a wife was not allowed to speak either for or against her husband. Doubtless this unpleasant matrimonial spectacle is likely enough to occur, but is it certain that such an event is one always to be deplored? A somewhat singular case in point may be cited which tends to a different conclusion; it is one of which all the details are known to me, and it took place only a few years ago. A man and his wife were sent to prison to await their trial for the murder of a child, who was the woman's own offspring, but stepson only to the man. When the case came into court the grand jury threw out the bill against the woman, but the man was tried for the crime. Among the persons present in the court to hear the trial was the lady visitor of the prison where the accused had waited for that momentous day. She had won the confidence of the woman, who was in a cruelly distressed state of mind, and who under a pledge of secrecy had told her the whole truth. She said that her husband had deliberately murdered the child, having always resented fiercely its presence in the house. The woman blamed herself—and most justly—for not having had the courage to oppose him when he went into the room where the little boy was alone, guessing accurately enough what his intention was. The sight of the dead child had filled her with a remorse which never left her, but it was only to her friend in the prison that she ever told it. The trial lasted long—the murderer, who expected to be convicted, looking ghastly with terror—but the judge ruled that the evidence was not sufficient against him, and he was acquitted, the one person in the court who knew the truth being of necessity silent, and so a great miscarriage of justice was accomplished. It seems fair to hold that justice to prisoners as well as to the public, who must be protected from evildoers, will alike be promoted by the passing of this new beneficent Act.

The conviction that the objections raised against it are groundless has been strongly supported by much that has taken place, even in the short time which has elapsed since the Act came into active operation. The majority of the prisoners who have been tried since then have elected to speak in their own defence. Amongst them was a man who attempted to prove an *alibi* by statements which the judge instantly detected to be absolutely false. He told the prisoner sternly that he might have been charged with perjury had he not been about to receive a heavy sentence in punishment of his crime.

